# Population genetic and field‐ecological analyses return similar estimates of dispersal over space and time in an endangered amphibian

**DOI:** 10.1111/eva.12479

**Published:** 2017-04-27

**Authors:** Ian J. Wang, H. Bradley Shaffer

**Affiliations:** ^1^ Department of Environmental Science, Policy, and ManagementUniversity of CaliforniaBerkeleyCAUSA; ^2^ Department of Ecology and Evolutionary BiologyUniversity of CaliforniaLos AngelesCAUSA; ^3^ La Kretz Center for California Conservation ScienceInstitute of the Environment and SustainabilityUniversity of CaliforniaLos AngelesCAUSA

**Keywords:** amphibian, conservation genetics, dispersal, effective population size, gene flow, landscape genetics, population connectivity

## Abstract

The explosive growth of empirical population genetics has seen a proliferation of analytical methods leading to a steady increase in our ability to accurately measure key population parameters, including genetic isolation, effective population size, and gene flow, in natural systems. Assuming they yield similar results, population genetic methods offer an attractive complement to, or replacement of, traditional field‐ecological studies. However, empirical assessments of the concordance between direct field‐ecological and indirect population genetic studies of the same populations are uncommon in the literature. In this study, we investigate genetic isolation, rates of dispersal, and population sizes for the endangered California tiger salamander, *Ambystoma californiense*, across multiple breeding seasons in an intact vernal pool network. We then compare our molecular results to a previously published study based on multiyear, mark–recapture data from the same breeding sites. We found that field and genetic estimates of population size were only weakly correlated, but dispersal rates were remarkably congruent across studies and methods. In fact, dispersal probability functions derived from genetic data and traditional field‐ecological data were a significant match, suggesting that either method can be used effectively to assess population connectivity. These results provide one of the first explicit tests of the correspondence between landscape genetic and field‐ecological approaches to measuring functional population connectivity and suggest that even single‐year genetic samples can return biologically meaningful estimates of natural dispersal and gene flow.

## 1 Introduction

Population genetic analyses of natural systems have become the empirical cornerstones of ecological and evolutionary genetics. The disciplines of ecological, conservation, and landscape genetics are immensely appealing because of their abilities to facilitate deeper understanding of the role of geographical and environmental features in structuring patterns of dispersal, genetic variation, and population demography (Lourenco, Alvarez, Wang, & Velo‐Anton, 2017; Storfer, Murphy, Spear, Holderegger, & Waits, 2010; Wang, Glor, & Losos, 2013). Population genetic methods have been implemented in a large number of empirical studies on a wide variety of systems (Hedrick, 2001; Storfer et al., 2010) and now constitute one of the most important approaches for efficiently quantifying population dynamics and microevolutionary processes in the wild. These methods frequently allow for the rapid assessment of population size, structure, and connectivity in natural systems and are especially valuable for species in which direct observations are difficult (Wang, Savage, & Bradley Shaffer, 2009) or that are of conservation concern (Sommer, McDevitt, & Balkenhol, 2013; van Strien et al., 2013). Although studies have shown them to be statistically powerful and to perform well under simulated conditions (Hedrick, 2001; Storfer et al., 2010; Wang, 2013), the reliability of indirect genetic analyses to match field‐ecological estimates of the population parameters of interest to conservationists and resource managers can only be evaluated by comparison with direct observational studies from complex systems in nature.

Unfortunately, field‐based and genetic estimates of important conservation parameters have only seldom been rigorously compared in an empirical framework (Jones, 2010; Richardson, Brady, Wang, & Spear, 2016), and the level and nature of correspondence between field mark–recapture and genetic estimates of dispersal have long been debated (Lowe & Allendorf, 2010; McKechnie, Ehrlich, & White, 1975; Wang, 2009a; Watts et al., 2006; Yu, Nason, Ge, & Zeng, 2010). In some studies, molecular estimates of gene flow exceeded those predicted from field observations (Jones, 2010; Wang, 2009a; Watts et al., 2006), a situation that has been termed “Slatkin's Paradox” (Koenig, Van Vuren, & Hooge, 1996; Marko & Hart, 2011; Yu et al., 2010). In others, inferred rates of gene flow were lower than expected based on the natural history of the study system (De Meester, Gómez, Okamura, & Schwenk, 2002; Uthicke & Benzie, 2003). Differences between such independent estimators can be explained in at least three ways: (i) high variance across time or space leading to unreliable estimates of parameter values from molecular or field studies based on single point estimates, (ii) low accuracy of one or both methods generating incongruence due to noise or error, or (iii) biological differences in what each class of methods is actually measuring. In the first two cases, differences between direct field studies and indirect genetic studies essentially result from statistical or methodological artefacts, while in the third, they result from each method measuring what are actually different population parameters even though both are meant to be indicators of the same biological process or property. For example, if dispersal is common but the reproductive success of dispersers is comparatively low, then direct field studies of “dispersal” should consistently estimate higher levels than “dispersal” estimates based on gene flow inferred by indirect genetic studies. In this case, both may be accurate, but they are (perhaps unintentionally) measuring different aspects of population connectivity.

Isolating the effects of these three potential explanations for differences between field‐ecological and genetic studies is extremely challenging and requires study systems that are well characterized and reliably return accurate estimates of demographic parameters from both methodologies. Pond‐breeding amphibians are well suited to this task. In these systems, dispersal behavior is often closely tied to breeding, generally occurs at low to moderate rates, and primarily takes place on local scales with few or no long‐distance dispersal events (Murphy, Dezzani, Pilliod, & Storfer, 2010; Shaffer & Trenham, 2005; Smith & Green, 2005; Wang et al., 2009). Additionally, because breeding occurs more or less synchronously in discrete ponds (Murphy et al., 2010; Smith & Green, 2005; Spear, Peterson, Matocq, & Storfer, 2005; Wang, 2012), breeding populations can be unambiguously sampled and delimited, reducing potential sampling error and allowing for the confident assignment of individuals to physical populations. Given these attributes, pond‐breeding amphibians constitute excellent test cases for exploring the reliability and repeatability of field and genetic methods to return accurate estimates of dispersal and effective population size.

In this study, we explicitly test the concordance between independent, field‐ecological and population genetic estimates of dispersal and population size when the data are drawn from large, multigeneration samples. We estimated dispersal rates, genetic structure, and population sizes in a pond‐breeding amphibian, the California tiger salamander, *Ambystoma californiense*. The species is well characterized ecologically and is listed as threatened or endangered under US federal law (US Endangered Species Act) and as threatened under California law (California Endangered Species Act). Adults typically breed and disperse between breeding populations only once or twice in their lifetimes and only during the restricted winter rainy season (Shaffer & Trenham, 2005; Trenham, Bradley Shaffer, Koenig, Stromberg, & Ross, 2000). Based on two decades of intensive field study, our understanding of their breeding biology suggests that dispersers and nondispersers are equally successful breeders (Shaffer & Trenham, 2005) and that dispersal occurs at moderate rates over local landscapes (Searcy, Gabbai‐Saldate, & Shaffer, 2013; Trenham, Koenig, & Shaffer, 2001; Wang et al., 2009).

We collected DNA samples from two breeding seasons separated by 6 years (approximately 1.5‐2 generations) in 1995 and 2001 from the same 12 breeding ponds on the Hastings Natural History Reservation and adjacent Oak Ridge Ranch (hereafter referred to as Hastings) in Monterey County, California. Earlier work from the same sites (Trenham et al., 2000, 2001) allowed us to directly compare the congruency of our genetic estimates of key population parameters with more traditional field‐ecological estimates based on mark–recapture methods conducted from 1995 to 1998. Our study design allowed us to compare genetic estimates across years and to rigorously assess the concordance of field‐ecological and genetic methods while controlling for sampling variance and biological factors that could produce discrepancies.

## 2 Materials and Methods

### 2.1 Study system and sampling

The California tiger salamander, *Ambystoma californiense*, is a pond‐breeding amphibian endemic to central California and is listed as threatened by the state of California and threatened or endangered in different parts of its range under the U.S. Endangered Species Act (Shaffer & Trenham, 2005). *Ambystoma californiense* breed in seasonal and, less frequently, permanent ponds that are free of fish and other non‐native predators. Aquatic larvae grow in these pools for 3–6 months, at which time they metamorphose and disperse into the surrounding terrestrial landscape. Fitness, including dispersal ability, is strongly tied to size at and time to metamorphosis (Searcy, Gray, Trenham, & Shaffer, 2014). Aside from a few weeks of breeding activity, they are primarily terrestrial and fossorial, residing in small mammal burrows (primarily California ground squirrel, *Otospermophilus beecheyi*, and Botta's pocket gopher, *Thomomys bottae*) which provide protection against predation and desiccation (Searcy & Shaffer, 2008; Shaffer & Trenham, 2005; Trenham & Shaffer, 2005). Although *A. californiense* have a maximum life span of about 11 years, they generally breed only once or twice during their lifetimes, and typically breed for the first time at 4 years of age (Trenham et al., 2000).

**Figure 1:**
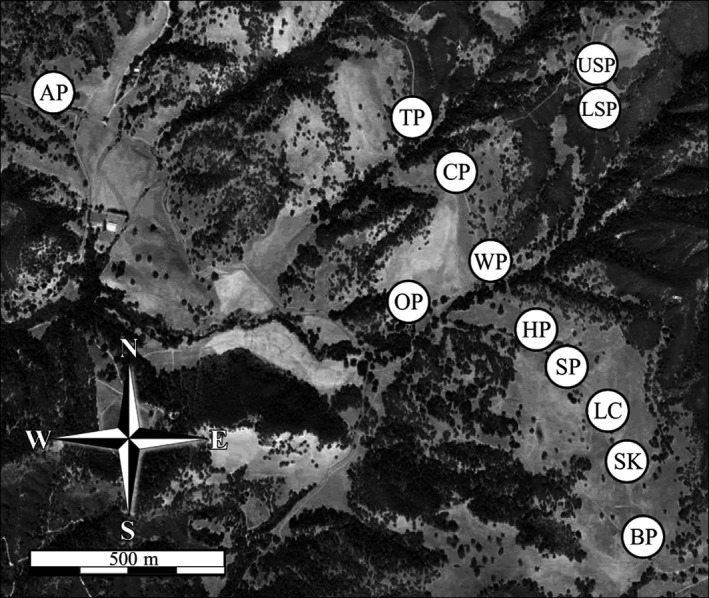
Sampled breeding ponds of *Ambystoma californiense* near the Hastings Natural History Reserve, Monterey County, CA, on a satellite imagery map showing vegetation and topographic relief. Pond acronyms as in Table 1

We conducted our research on an intact set of natural and modified vernal pools adjacent to the Hastings Natural History Reservation on Oak Ridge Ranch, Monterey County, California (Table 1; Figure 1). Late‐stage larvae were captured by seining, tissues were sampled as small tail‐clips and preserved in 95% ethanol, and larvae were immediately released at the point of capture with no apparent harm to the animals (Polich, Searcy, & Shaffer, 2013). We collected a total of 716 samples from 12 breeding ponds: 360 in 1995 and 356 in 2001. We were unable to sample two of the pools in 1995 and four others in 2001, resulting in six ponds sampled in both years (Table 1). These collections were made using minimally invasive procedures following protocols approved by the UC Davis Institutional Animal Care and Use Committee (IACUC) and under scientific permits issued by the State of California Department of Fish and Wildlife (SC‐8436) and the United States Department of the Interior Fish and Wildlife Service (TE‐094642).

**Table 1:** Breeding pond characteristics and effective population size [*N*_e_] estimates for localities of *Ambystoma californiense* at Hastings, Monterey County, California. For each sampled population, we show the number of tissue samples [*N*] collected in 1995 and 2001, the pond area [m^2^], rodent burrow density [burrows/400 m^2^], and number of breeding adults from field estimates (*N*_b_[Field]), followed by the mean and 95% confidence interval (in parentheses) for *N*_e_ for each sampling year (*N*_e_[1995] and *N*_e_[2001]) and based on a multiyear temporal method (*N*_e_[Temp]). Area, burrow, and *N*_b_(Field) data are from (Trenham et al., 2001)

Acronym	Pond Name	*N*(1995)	*N*(2001)	Area	Burrows	*N*_b_(Field)	*N*_e_(1995)	*N*_e_(2001)	*N*_e_(Temp)
BP	Blomquist Pond	38	41	700	26	67.6	18 (14–22)	14 (9–19)	21 (12–30)
SK	Sink Pond	37	–	370	13	11.8	12 (7–17)	–	–
LC	Laguna Conejo	36	–	3660	7	278.7	43 (31–55)	–	–
HP	Hidden Pond	37	–	1250	14	3.3	29 (20–37)	–	–
SP	Salamander Pond	33	–	640	9.8	7.4	17 (12–22)	–	–
WP	Windmill Pond	36	43	470	5.5	58.4	8 (4–12)	5 (2–8)	11 (5–16)
CP	Creche Canyon Pond	40	45	360	6.3	20.9	8 (5–12)	7 (4–9)	12 (6–18)
TP	Triangle Pond	38	40	460	12	41.1	9 (5–13)	9 (4–13)	12 (9–15)
AP	Ardillas Pond	–	50	400	–	5.3	–	7 (2–12)	–
USP	Upper Steep Pond	40	48	–	–	–	13 (7–19)	19 (12–26)	22 (13–31)
LSP	Lower Steep Pond	–	49	–	–	–	–	10 (5–15)	–
OP	Old Road Pond	25	40	–	–	–	15 (8–21)	12 (7–17)	18 (10–26)

### 2.2 Genotyping

We digested tissues in lysis buffer with Proteinase K and purified genomic DNA using a standard ethanol precipitation. Extracted samples were diluted to 10 ng/μl and used as template in PCR reactions for 15 tetra‐nucleotide microsatellite loci (Savage, 2008), which have traditionally been the preferred genetic markers for landscape and conservation genetics studies (Thomson, Wang, & Johnson, 2010; Wang, 2011). Forward primers for each PCR were labeled with a 5' fluorescent tag (6‐FAM, NED, VIC, or PET) for visualization. We amplified loci individually and ran PCR products on an ABI 3730 Genetic Analyzer. Fragments were sized with LIZ‐500 size standard, collected with genescan, and scored with STrand. We used micro‐checker (Van Oosterhout, Hutchinson, Wills, & Shipley, 2004) to identify potential scoring errors, the presence of null alleles, linkage disequilibrium, and departures from Hardy–Weinberg equilibrium (HWE). To test for repeatability in microsatellite scoring, we repeated all steps from amplification through scoring on a set of 48 samples (6.7% of all samples). We have deposited the resulting microsatellite genotype data in the Dryad Data Repository.

### 2.3 Genetic structure and dispersal

We calculated pairwise values of F_ST_ between ponds for each sampling year (1995, 2001) and F_ST_ between years for each pond (each pond in 1995 compared to itself in 2001), as basic metrics of population structure and temporal differentiation, using GenAlEx (Peakall & Smouse, 2005). We performed a Mantel test to quantify the correlation in F_ST_ between years for the subset of six ponds sampled in both years using the “vegan” package in R (Oksanen, Kindt, Legendre, Ohara, & Stevens, 2007).

To estimate dispersal between populations, we used a genetic assignment method implemented in bayesass+ (Wilson & Rannala, 2003). bayesass+ uses a fully Bayesian MCMC resampling approach to estimate recent, asymmetrical dispersal rates between populations (Berry, Tocher, & Sarre, 2004; Paetkau, Slade, Burden, & Estoup, 2004; Wilson & Rannala, 2003) and also calculates a confidence interval for results that would be returned from uninformative data (typically those that do not contain sufficient variation to estimate dispersal with high confidence (Wilson & Rannala, 2003; Pearse & Crandall, 2004). Whereas coalescent‐based methods return estimates of long‐term rates of dispersal, as a genetic assignment method, bayesass+ provides estimates of recent or contemporary dispersal rates (Pearse & Crandall, 2004). Thus, by estimating recent and asymmetric rates of dispersal, bayesass+ provides genetic estimates that are suitable for comparison with field estimates based on sampling over short timescales (e.g. Trenham et al., 2001). We performed one run with five million generations, discarded the first two million (40%) as burn‐in, and sampled the remaining chain every 2000 generations using default parameter settings.

To compare our molecular estimates of dispersal to field‐based estimates, we tested the fit of the rates we inferred from bayesass+ to the regression function estimated by Trenham et al. (2001), based on among‐pond mark–recapture studies for the same ponds at Hastings from 1995 to 1998. We calculated the coefficient of determination (R^2^) from the sum of squares of the residuals between our points and the dispersal function estimated from mark–recapture field data, y = 0.264e^−0.0028x^, where *y* is the dispersal rate or probability and *x* is the distance between ponds (Trenham et al., 2001). We tested the significance of this R^2^ value using an *F* test to determine whether our data constituted a significant fit to the ecological dispersal function from Trenham et al. (2001). Essentially, this tests whether the disparity of our observed points from their expected values based on the dispersal function indicates a significant deviation from the mark–recapture based expectations. For this analysis, we pooled dispersal rates across our two sampling years (1995, 2001) to increase statistical power after testing for similar population structure between years and to more closely match the field estimates since Trenham et al. (2001) pooled estimates across multiple years (1995–1998) in their study. All statistical analyses were performed in R (R Core Team 2015). We also used the “lm” and “nls” functions of the “stats” package in R to fit and compare simple linear regressions and exponential regressions of dispersal rate as a function of distance between ponds. We implemented negative exponential regression to allow us to compare our results with those of Trenham et al. (2001) and because negative exponential curves are often realistic models of dispersal (Austerlitz et al., 2004; Kot, Lewis, & van den Driessche, 1996; Trenham et al., 2001).

### 2.4 Effective population size estimation

To estimate effective population sizes of *A. californiense* in each of the sampled breeding ponds, we used the sibship assignment (SA) method implemented in colony (Wang, 2009b). This method first determines the probabilities of all pairs of samples drawn from a population being full‐sibs, half‐sibs, or nonsibs based on multilocus microsatellite data. These assignment probabilities are then used to fit a predictive equation that relates assignment probabilities to *N*_e_ given a randomly sampled, single cohort; importantly, it does not require random mating, and it accounts for both genetic and sampling variance in its estimators (Wang, 2009b). This analysis was performed on each breeding pond from each year independently. We also estimated *N*_e_ using a temporal method that utilizes changes in allele frequencies between years. This method estimates the most likely *N*_e_ for a population that would result in the observed allele frequency changes under a model of drift and migration (Wang & Whitlock, 2003; Waples, 1989). These temporal estimates were performed in the program MLNE (Wang & Whitlock, 2003).

We performed regression of *N*_e_ against pond area and rodent burrow density to examine correlations between these variables. Pond area has been shown to be a strong predictor of *N*_e_ in this species on a different landscape (Wang, Johnson, Johnson, & Shaffer, 2011), and we predicted that the same pattern would be the case at Hastings. Pond area measurements and burrow density (burrows/400 m^2^) were log‐transformed, and bivariate linear regressions were performed using the “lm” function in R. The field data on burrows and pond area were published previously (Trenham et al., 2001). In that study, pond areas were measured by aerial imagery and burrows were counted along four 1 m wide transects extending 100 m in each of the cardinal directions from the edge of each pond.

To estimate the correlation between *N*_e_ estimates obtained from the SA genetic method and a field‐based mark–recapture (MR) method, we used simple linear regression in R. We acquired MR estimates of *N*_e_ from the numbers of breeding adult males and females observed by Trenham et al. (2001), using Wright's (Wright, 1938) method to estimate the sex ratio effective size based on the equation

$$N_{e}=\frac{4N_{m}N_{f}}{N_{m}+N_{f}},$$

where *N* is the number of adult male (m) or female (f) individuals.

## 3 Results

### 3.1 Genotyping

All 15 of our microsatellite loci were highly polymorphic, containing from 6 to 18 alleles with an average of 11.8 alleles per locus. micro‐checker (Van Oosterhout et al., 2004) did not indicate the presence of null alleles, scoring errors, or linkage disequilibrium, but did detect deviations from HWE in a few loci in some populations. Because none of these loci showed significant deviations in most populations, we did not exclude any of them from the analyses. We could not unambiguously score 3.6% of the genotypes, and these were coded as missing data. The 48 samples that were amplified and scored twice produced identical results in each trial.

### 3.2 Genetic structure and dispersal

**Figure 2:**
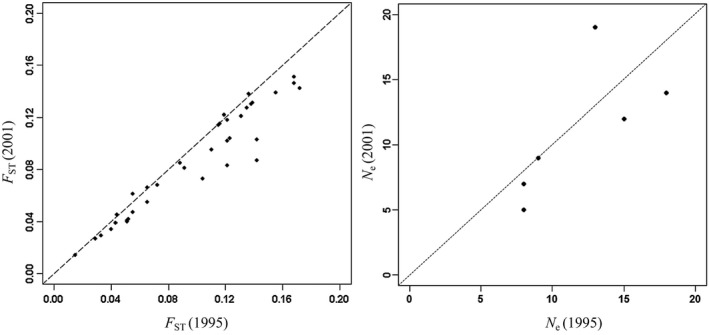
Scatterplots of (left) pairwise F_ST_ between ponds in 1995 (*x*‐axis) and 2001 (*y*‐axis) and (right) *N*_e_ in 1995 and 2001. The dashed line shows where equal values would lie (y=x)

We found low to moderately high levels of genetic structure among the 12 breeding ponds at Hastings (Table 2), ranging from F_ST_ = 0.014 to 0.202 (mean pairwise F_ST_ = 0.107 ± 0.051) in 1995 and from F_ST_ = 0.009 to 0.210 (mean pairwise F_ST_ = 0.130 ± 0.048) in 2001. Values of pairwise F_ST_ between breeding ponds (Table 2; Figure 2) were quite similar between each year of sampling for those ponds that were sampled in both years (Mantel's r = 0.933, *p* < .002). We also detected low levels of genetic differentiation between years within breeding ponds, ranging from F_ST_ = 0.002 to 0.044 (mean F_ST_ = 0.019 ± 0.016; Table 2).

**Table 2:** Estimates of pairwise F_ST_, based on 15 highly variable microsatellite loci. Values below the diagonal are from sampling performed in 1995 and above the diagonal are from 2001

	BP	SK	LC	HP	SP	WP	CP	TP	AP	USP	LSP	OP
BP	**0.009**	–	–	–	–	0.132	0.168	0.172	0.194	0.210	0.203	0.104
SK	0.085	**–**	–	–	–	–	–	–	–	–	–	–
LC	0.081	0.014	**–**	–	–	–	–	–	–	–	–	–
HP	0.104	0.039	0.034	**–**	–	–	–	–	–	–	–	–
SP	0.102	0.047	0.026	0.027	**–**	–	–	–	–	–	–	–
WP	0.126	0.068	0.055	0.042	0.061	**0.007**	0.082	0.093	0.144	0.125	0.129	0.032
CP	0.153	0.139	0.132	0.124	0.115	0.073	**0.031**	0.121	0.163	0.110	0.109	0.061
TP	0.140	0.146	0.138	0.127	0.122	0.087	0.083	**0.044**	0.151	0.128	0.133	0.088
AP	–	–	–	–	–	–	–	–	**–**	0.176	0.169	0.136
USP	0.202	0.197	0.184	0.161	0.200	0.131	0.105	0.111	–	**0.002**	0.009	0.157
LSP	–	0.194	0.186	0.172	0.189	0.126	0.119	0.116	–	0.037	**–**	0.148
OP	0.118	0.089	0.077	0.045	0.052	0.048	0.071	0.104	–	0.130	**–**	**0.020**

F_ST_ values comparing 1995 and 2001 for the same population are along the diagonal in bold. Pond acronyms correspond to Figure 1 and Table 1. Missing values (‐) are because some populations could not be sampled in both years.

**Figure 3:**
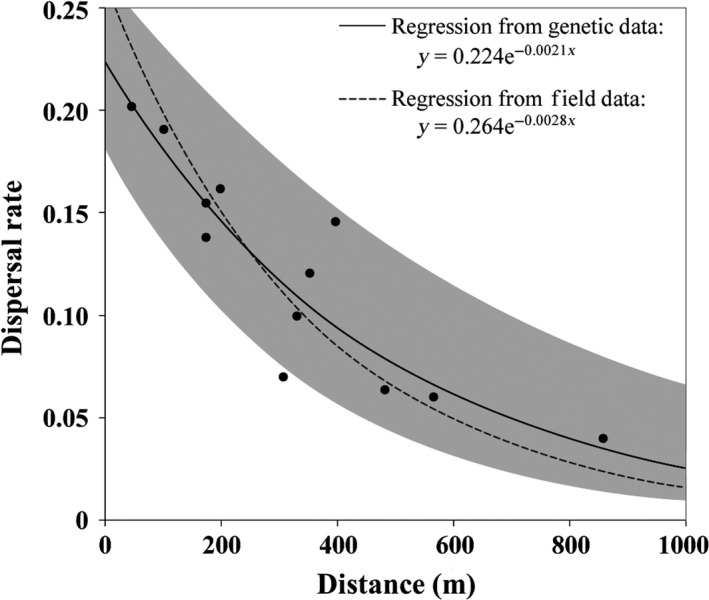
Estimates of dispersal rates between breeding ponds of *Ambystoma californiense*, plotted as the distance between ponds vs their pairwise dispersal rate (Table 3). Also shown are the regression line based on these genetic data (solid) and the regression line based on field data (dashed) from a previously published study of the same set of ponds (Trenham et al., 2001). Gray shading indicates the 95% confidence interval around the regression line based on genetic data

Estimation of gene flow in bayesass+ indicated that dispersal between some population pairs is common (Table 3). The analysis indicated significant rates of dispersal ranging from *m *=* *0.042 to 0.202 (Table 3). These values indicate the proportion (*m*) of sampled larvae from each breeding pond with immigrant ancestry in the current generation. These rates were a significant fit (R^2^ = .755; *p* = .045; Figure 3) to the dispersal probability function estimated by Trenham et al. (2001) based on field mark–recapture data (Figure 3). Field estimates (Trenham et al., 2001) yielded *y *=* *0.264*e* ^−0.0028*x*^, where *y* is the dispersal rate or probability and *x* is the distance between ponds. The dispersal probability function inferred from our results was *y *=* *0.224*e* ^−0.0021*x*^.

**Table 3:** Dispersal rates inferred under a genetic assignment method implemented in BayesAss+ (Wilson & Rannala, 2003) from a source pond to a destination pond in either 1995 or 2001. The mean dispersal rate is followed by the 95% confidence interval in parentheses. Nonsignificant dispersal rates are not shown; pond acronyms are as in Table 1

Year	Source → Destination	Dispersal Rate
1995	LC → SK	0.191 (0.175–0.219)
1995	LC → SP	0.155 (0.131–0.173)
1995	SP → LC	0.138 (0.127–0.149)
1995	SP → HP	0.162 (0.142–0.181)
1995	WP → CP	0.121 (0.106–0.137)
1995	BP → SK	0.064 (0.044–0.084)
1995	WP → LC	0.042 (0.033–0.052)
1995	TP → OP	0.060 (0.050–0.069)
1995	WP → OP	0.100 (0.089–0.111)
2001	CP → WP	0.146 (0.118–0.172)
2001	TP → CP	0.070 (0.058–0.082)
2001	USP → LSP	0.202 (0.171–0.232)

### 3.3 Effective population size estimation

The sibship assignment (SA) method (Wang, 2009b) indicated that effective population sizes (*N*_e_) were relatively small in each of the breeding ponds at our study site (Table 1). *N*_e_ estimates for 10 breeding ponds ranged from 8 to 43 effective breeders in 1995 (mean *N*_e_ = 17.2 ± 11.0) and from 5 to 19 (six ponds) in 2001 (mean *N*_e_ = 10.4 ± 4.5). The temporal method, which could only be applied to the six ponds sampled in both years, returned similarly small, but consistently greater estimates of *N*_e_, ranging from 11 to 22 effective breeders (mean *N*_e_ = 16.0 ± 4.9). The effective numbers of breeders in each population estimated by a field‐based mark–recapture (MR) method (*N*_b_ Field; Table 1) and *N*_e_ inferred by SA in 1995 were correlated (r^2^ = .460, *p* = .039), although this relationship was driven by a single large population in both methods (population LC). When this population was removed from the analysis, MR and SA estimates were not significantly correlated (r^2^ = −.023, *p* = .395). Likewise, we did not detect significant correlations between MR and SA methods for 2001 after removing LC, nor between MR and the temporal method (*N*_e_ Temporal; Table 1).

We detected strong linear correlations between log‐transformed pond area and *N*_e_ inferred by SA averaged across years (slope = 15.90, r^2^ = .945, *p* < .001, *N* = 9). We also detected a significant correlation between pond area and *N*_e_ estimated by SA for 1995 (slope = 15.49, r^2^ = .956, *p* < .001, *N* = 8) and positive though not statistically significant (at the *p* ≤ .05 level) correlations with *N*_e_ estimated by SA for 2001 (slope = 11.02, r^2^ = .543, *p* = .096, *N* = 5) and *N*_e_ inferred by the temporal method (slope = 14.81, r^2^ = .633, *p* = .131, *N* = 4). Our power to detect a significant relationship in the latter two cases was constrained by low sample sizes (five and four populations, respectively). We found no correlation between rodent burrow density and *N*_e_ for any year or estimation method (*p* > .40 in all cases), suggesting that upland habitat retreats within 100 m of a breeding site are not limiting population size.

## 4 Discussion

Landscape and conservation genetics are powerful research programs that promise to provide insights into patterns of movement, habitat preferences, and population sizes of organisms in nature that are otherwise difficult to obtain (Hedrick, 2001; Spear, Balkenhol, Fortin, Mcrae, & Scribner, 2010; Storfer et al., 2007; Wang, 2010). For many animals, it may be easier, cheaper, and potentially less stressful to capture, sample, and release members of a population and conduct straightforward genetic analyses than to conduct multiyear, mark–recapture studies. For endangered taxa like the California tiger salamander, it may also be the most efficient and least intrusive way to rapidly collect critical data for management and recovery (US Fish and Wildlife Service 2015). Although we do not advocate replacing field studies with molecular ones, we do feel that in many cases the molecular approach may provide fast, inexpensive, and accurate insights that complement the deep knowledge gained from long‐term field studies. Given the potential advantages of molecular approaches, in isolation and particularly when combined with field programs, and their value for conservation and management planning, a critical question is how reliably they measure key population parameters compared to well‐designed field studies of the same variables.

Our multigeneration study produced consistent results across years (1995 and 2001) for estimates of effective population sizes (Table 1) and between‐site genetic differentiation (Table 2; Figure 2). For the six ponds sampled in both years, estimates of *N*_e_ varied by just a few individuals (≤6) between years, and the confidence intervals for both years showed broad overlap (Table 1). Pairwise estimates of genetic differentiation (F_ST_) were also very similar in both years for these populations, and we found relatively little genetic differentiation between years within each population (F_ST_ ≤ 0.044; Table 2). These results are not surprising, given the relatively short time between samples; whether effective population sizes and genetic differentiation stay consistent across longer temporal scales remains to be seen. Similarly, with only six ponds resampled between years, our results are limited from informing us about whether temporal consistency is observed across broader spatial scales or whether populations experiencing different conditions might vary more through time. So far, few landscape and conservation genetics studies have examined the same populations across time (Richardson et al., 2016; Wang & Bradburd, 2014). For threatened and endangered species, especially those with patchy distributions, obtaining sufficient sampling from multiple years may present a challenge, but hopefully more studies contributing to these efforts in the future will provide valuable information on the temporal stability of population demographics and dynamics in various natural systems, including endangered species.

The consistency of population parameter estimates across years from genetic methods justifies comparing these estimates to more traditional, multiyear field‐based estimates of the same parameters. We found a strong similarity between our molecular estimates of dispersal across years and field‐based ecological estimates from a previously published study on the same landscape (Trenham et al., 2001). Our estimates of dispersal were a significant and remarkably close fit to the dispersal probability function based on Trenham et al.'s (2001) multiyear, mark–recapture data (R^2^ = .589; *p* = .045; Figure 3), indicating that these very different strategies returned highly concordant estimates of dispersal. There are many reasons, both statistical and biological, why molecular and field‐based estimates of dispersal may differ (Jones, 2010; Lowe & Allendorf, 2010; Yu et al., 2010). On the statistical side, error in each estimation procedure, sampling variance due to incomplete sampling of breeding populations, and inadequate sample sizes could all contribute. On the biological side, field‐based estimates typically tally all dispersal events between populations (as was done by Trenham et al., 2001), while molecular estimates of offspring only include dispersal events that result in successful reproduction. These statistical and biological issues may lead to differences between molecular and field‐based estimates, but they need not.

Here, we found that genetic and field‐based approaches provide reasonably congruent estimates, suggesting that both approaches can play valuable roles in effective conservation and management decisions for assessing and maintaining population connectivity. Obviously, this is not always the case, and several studies in other systems have found large differences between field‐ecological and genetic methods, particularly in species with passive or long‐distance dispersal, like flying insects (Mallet, 2001), aquatic invertebrates (De Meester et al., 2002; Uthicke & Benzie, 2003), and plants (Jones, 2010; Yu et al., 2010). Although we cannot conclusively say why our study returned such similar results across methodologies while others did not, the specific type of dispersal mechanism may contribute to the likelihood of field and genetic estimates returning congruent results. In particular, it may be that for systems in which dispersal takes place over small distances among well‐sampled sites and in which dispersers and residents have an equal likelihood of successful reproduction, the two approaches tend to produce reliable, commensurate results. These studies also estimated dispersal based on metrics of genetic differentiation (F_ST_), rather than methods that explicitly estimate dispersal from genetic data, and it is possible that incongruence between field and genetic estimates may be observed more frequently in genetic datasets that capture long‐term patterns of dispersal. It may also be important that systems have relatively consistent rates of dispersal through time or that genetic and field studies are conducted during the same time periods in order to detect concordant results between methodologies and that when point estimates are drawn from different time periods we should expect greater discrepancies simply due to random fluctuations. Additional case studies will be necessary to fully evaluate these possibilities.

In contrast to the concordance between estimates of dispersal, we only found a significant correlation between field‐based and genetic estimates of population sizes when a single, relatively large outlier population (LC) was included in the analysis. After removing population LC, the correlation across roughly similar‐sized ponds was not significant. Although the numbers of individuals we sampled per population were sufficient to generate reliable estimates of *N*_e_ (Hale, Burg, & Steeves, 2012), our power to detect significant relationships between field and genetic estimates of population sizes was limited by the relatively small number of ponds that we were able to sample and by the relatively small range in pond area. Hence, we cannot exclude the possibility that broader sampling could yield compatible estimates from field and genetic methods.

As with dispersal rates, a number of statistical and biological reasons could explain any incongruence between molecular and field‐based estimates of population sizes (Jehle, Arntzen, Burke, Krupa, & Hödl, 2001; Schmeller & Merilä, 2007). The most compelling are probably biological, because genetic estimates of *N*_e_ can be significantly lower than census estimates if there is reproductive skew among individuals, if a population has gone through a bottleneck, or if a population has experienced substantial genetic drift in isolation (Charlesworth, Charlesworth, & Barton, 2003; Nunney & Elam, 2002; Schmeller & Merilä, 2007). For *A. californiense,* reproductive skew may contribute to the observed differences, given that reproductive success varies substantially among males in other ambystomatid salamanders (Gopurenko, Williams, & DeWoody, 2007; Myers & Zamudio, 2004). This explanation can account for genetic estimates of *N*_e_ that are consistently lower than field‐ecological estimates, as is generally the case here, but it does not predict a lack of correlation between estimates. Given the consistency of our genetic estimates of *N*_e_ over a six‐year time span (Table 1) and the well‐established variability in the number of salamanders that breed across years both on this landscape (Trenham et al., 2000) and in other systems (Pechmann et al., 1991), these results suggest that although the total census number of *A. californiense* that show up to breed each year fluctuates, the actual number of reproductively successful adults in this system remains relatively consistent through time. From a management and recovery perspective, these results suggest that although both are informative, molecular estimates of *N*_e_ may return more meaningful numbers for tracking recovery and stability of populations over time. Population stabilization or growth, generally over decadal timescales, is a key recovery criterion for down‐ or delisting under the US Endangered Species Act, and molecular estimates of *N*_e_ appear to provide meaningful, repeatable estimates of actual breeding, rather than potential breeding based on census numbers of adults at a breeding site. This was recently suggested in a recovery plan for the Santa Barbara Distinct Population segment of *A. californiense* (U.S. Fish and Wildlife Service, 2015), and these results support that recommendation.

## 5 Conclusions

Overall, our results indicate that genetic assessments of migration and effective population sizes in natural systems are reliable and informative, especially when accompanied by complementary knowledge of field natural history. For example, in pond‐breeding amphibians, based on our knowledge of their reproductive biology, we expect that gene flow will be proportional to total dispersal among populations. In fact, for the California tiger salamander, the patterns of dispersal and population connectivity inferred from our genetic data were remarkably congruent with those based on field observations, suggesting these are reliable reflections of ongoing ecological processes. In a similar vein, past work demonstrates that pond area is an important component of population size in this species (Wang et al., 2011), a result that we also recovered here on an independent, ecologically different landscape. Moreover, our results raise the interesting possibility that when extensive field‐ecological and genetic analyses of population parameters disagree, these differences may result from interesting biological properties of the study organism. The lack of correlation between field census size estimates and genetic estimates of effective population size (*N*_e_), coupled with consistent estimates of *N*_e_ across years but high variability in census sizes (Trenham et al., 2001), suggests that regardless of the total number of adults arriving in breeding ponds, the number of reproductively successful breeders is close to stationary through time.

Maintaining functional population connectivity and effective population sizes are critical challenges in conservation, especially under scenarios of environmental change, habitat loss, and fragmentation (Hedrick, 2001; Sommer et al., 2013; van Strien et al., 2013). Genetic analyses have and should continue to play a major role in efforts to quantify and conserve metapopulation dynamics, which are key elements of long‐term sustainability (Marsh & Trenham, 2001; Taylor, 1990). Additional comparative studies will be necessary to reveal the relationships between ecological and genetic parameter estimates for a range of species with different dispersal abilities, breeding strategies, and life histories.

## Data Archiving Statement

Data available from the Dryad Digital Repository: https://doi.org/10.5061/dryad.tk751
